# SEGEL: A Web Server for Visualization of Smoking Effects on Human Lung Gene Expression

**DOI:** 10.1371/journal.pone.0128326

**Published:** 2015-05-26

**Authors:** Yan Xu, Brian Hu, Sammy S. Alnajm, Yin Lu, Yangxin Huang, Diane Allen-Gipson, Feng Cheng

**Affiliations:** 1 Department of Pharmaceutical Science, College of Pharmacy, University of South Florida, Tampa, Florida, United States of America; 2 Department of Biology, Faculty of Mathematics and Natural Sciences, University of Cologne, Cologne, Germany; 3 Department of Department of Epidemiology and Biostatistics, College of Public Health, University of South Florida, Tampa, Florida, United States of America; 4 Division of Allergy and Clinical Immunology, College of Medicine, University of South Florida, Tampa, Florida, United States of America; Queen's University Belfast, UNITED KINGDOM

## Abstract

Cigarette smoking is a major cause of death worldwide resulting in over six million deaths per year. Cigarette smoke contains complex mixtures of chemicals that are harmful to nearly all organs of the human body, especially the lungs. Cigarette smoking is considered the major risk factor for many lung diseases, particularly chronic obstructive pulmonary diseases (COPD) and lung cancer. However, the underlying molecular mechanisms of smoking-induced lung injury associated with these lung diseases still remain largely unknown. Expression microarray techniques have been widely applied to detect the effects of smoking on gene expression in different human cells in the lungs. These projects have provided a lot of useful information for researchers to understand the potential molecular mechanism(s) of smoke-induced pathogenesis. However, a user-friendly web server that would allow scientists to fast query these data sets and compare the smoking effects on gene expression across different cells had not yet been established. For that reason, we have integrated eight public expression microarray data sets from trachea epithelial cells, large airway epithelial cells, small airway epithelial cells, and alveolar macrophage into an online web server called SEGEL (Smoking Effects on Gene Expression of Lung). Users can query gene expression patterns across these cells from smokers and nonsmokers by gene symbols, and find the effects of smoking on the gene expression of lungs from this web server. Sex difference in response to smoking is also shown. The relationship between the gene expression and cigarette smoking consumption were calculated and are shown in the server. The current version of SEGEL web server contains 42,400 annotated gene probe sets represented on the Affymetrix Human Genome U133 Plus 2.0 platform. SEGEL will be an invaluable resource for researchers interested in the effects of smoking on gene expression in the lungs. The server also provides useful information for drug development against smoking-related diseases. The SEGEL web server is available online at http://www.chengfeng.info/smoking_database.html.

## Introduction

Data from the World Health Organization (WHO) show that cigarette smoking is one of the biggest threats to public health. Cigarette smoking caused 100 million deaths over the 20th century throughout the world [1]. About 19.5% of American adults are current tobacco users in the United States [2]. Cigarette smoking caused more than 440,000 fatalities per year [3], and smoke-related diseases cost 96 billion dollars in direct medical care expenses and 97 billion dollars in lost productivity in the United States [4]. In the human body, the primary organ that is affected by smoking is the lung. The toxins from cigarette smoke can directly injure lung structures and cause permanent airway damage. In present smokers, nearly 73% of smoking-related problems are serious lung diseases including COPD and lung cancer. Cigarette smoking is a major risk factor for COPD [5–7] that is the third leading cause of mortality and morbidity in the United States. Smoking is accountable for approximately 80–90% of COPD deaths and 90% of the lung cancer deaths in the United States.

Precise detection of changes in gene expression between smokers and nonsmokers is crucial to the understanding of the molecular mechanisms of smoking-induced lung diseases. Recently, high-throughput techniques such as expression microarray have been applied to detect smoking effects on gene expression in human lung cells. These research projects generated valuable data and provided new insights into the effects of tobacco smoke. However, a user-friendly web server that allows scientists to fast query the gene expression levels from these data sets and compare smoking effects on gene expression across different data sets had not been established.

In this paper, we report an online web server called SEGEL (Smoking Effects on Gene Expression of Lung) for easy and fast visualization of smoking effects on human lung gene expression. The web server integrates 362 samples collected from eight public expression microarray data sets from trachea epithelial cells, large airway epithelial cells, small airway epithelial cells, and alveolar macrophage. Gene expression patterns of regular smokers and nonsmokers across these cells can be queried by gene symbols. Sex difference in response to smoking is also shown. The correlation coefficients between the gene expression and cigarette smoking consumption (the number of packs of cigarettes consumed per year) were also calculated and are shown in the web server. The current version of SEGEL contains around 42,400 annotated gene probe sets represented on the Affymetrix Human Genome U133 Plus 2.0. SEGEL will be an invaluable tool and resource for scientists interested in the effects of smoking on lung gene expression. The web server can be used to identify reliable molecular signatures for drug discovery against smoking-related diseases. The SEGEL web server is available online at http://www.chengfeng.info/smoking_database.html

## Materials and Methods

362 samples in eight published microarray sets (Shown in Table 1) from the NCBI Gene Expression Omnibus (GEO) repository were integrated in the SEGEL to compare gene expression of smokers and nonsmokers in alveolar macrophage, small airways epithelial cells, large airways epithelial cells, and trachea epithelial cells. Detailed information of these 362 samples is provided in the Supplementary Information (S1 Table).

**Table 1:** Eight microarray datasets integrated in the SEGEL web server.

Data set	Cell	NCBI GEO entry ID	#Nonsmokers	#Smokers	Cigarette intake (Packs-year)
Trachea	Trachea Epithelial Cells	GSE13933	26	19	28±16
LargeAirway1	Large Airway Epithelial Cells	GSE10006	9	20	36±19
LargeAirway2	Large Airway Epithelial Cells	GSE10135	14	18	18±13
SmallAirway1	Small Airway Epithelial Cells	GSE4498	12	10	33 ± 7
SmallAirway2	Small Airway Epithelial Cells	GSE13933	23	19	27 ± 15
SmallAirway3	Small Airway Epithelial Cells	GSE30063	60	73	NA
Macrophage1	Alveolar Macrophage	GSE8823	11	13	36 ± 6
Macrophage2	Alveolar Macrophage	GSE13896	13	22	22 ± 14

### Trachea epithelial cells

The trachea is a tube that connects mouth/nose to the lungs and is protected by an epithelial cell layer. The trachea epithelium is extremely vulnerable to cigarette smoking. One microarray data set GSE13933 was chosen to investigate gene expression of trachea epithelium [8]. This data set includes 26 healthy nonsmokers and 19 regular smokers (28±16 packs-year).

### Large airway epithelial cells

Large airways, or bronchi, are airways that are divided from the trachea. Cigarette smoking could possibly be connected with the growth of cancer in the large air epithelium. Two data sets were evaluated in the large airways. GSE10006 [9] and GSE10135 [10] were chosen in SEGEL to evaluate gene expression in large airway epithelial cells. The first data set includes nine healthy nonsmokers and 20 regular smokers (36±19 packs-year). The second data set, GSE10135, includes 14 healthy nonsmokers and 18 regular smokers (18±13 packs-year).

### Small airway epithelial cells

Small airway epithelium was considered as the first site of lung pathology induced by cigarette smoking. Three GEO data sets, GSE4498, GSE13933 [8] and GSE30063 [11], were selected in SEGEL. The first data set includes 12 healthy nonsmokers and 10 regular smokers (33 ± 7 packs-year). The second data set includes 26 healthy nonsmokers and 19 regular smokers (27 ± 15 pack/year). The third data set includes 60 healthy nonsmokers and 73 regular smokers.

### Alveolar macrophage

An alveolar macrophage is a type of macrophage that is located on the epithelial surface area of bronchi alveoli. Alveolar macrophages supply initial protection against infectious, toxic, or sensitive contaminants from smoking. Alveolar macrophage appears to play an important role in initiating and sustaining COPD progression [12]. Two microarray data sets GSE8823 [13] and GSE13896 [14] were selected in SEGEL. In the first data set, alveolar macrophages were obtained from bronchoalveolar lavage of 11 nonsmokers and 13 regular smokers (36 ± 6 pack-year). In the second data set, alveolar macrophages were obtained from 13 nonsmokers and 22 regular smokers (22 ± 14 pack-year).

### Data normalization and analysis

All 362 samples in SEGEL were profiled with the Affymetrix HG-U133 Plus 2.0 GeneChip microarray platform that features comprehensive coverage of the human transcriptome. The current version of SEGEL contains 42,400 annotated gene probe sets with official gene symbols. The RMAexpress program was used to normalize raw microarray data and to summarize expression of the probe sets using the default settings: RMA background correction, quantile normalization, and log2-transformation. A boxplot (S1 Fig) was used to check whether the signal intensity distributions of samples from different data sets are similar.

Comparisons of the mean expression levels between smokers and nonsmokers were carried out by an unpaired t-test. All figures in SEGEL were plotted using R.

## Results and Discussion

SEGEL is a php-based online web server for easy and fast visualization of smoking’s effects on human lung gene expression. 362 samples in eight published microarray sets (shown in Table 1) from the NCBI Gene Expression Omnibus (GEO) repository were integrated in SEGEL to compare gene expression of smokers and nonsmokers in alveolar macrophage, small airways epithelial cells, large airways epithelial cells, and trachea epithelial cells. Analyzed microarray data are stored in a MySQL relational database.

**Fig 1:**
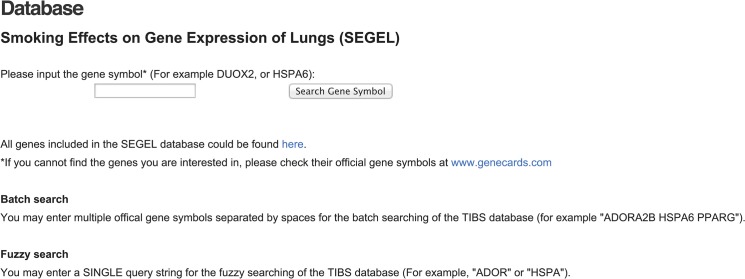
The query interface of the SEGEL web server. The input to the web server is official gene symbols. All probe sets representing the queried gene symbol will be shown; each probe set is reported in one PDF file. ADORA2B gene was used as an example to query SEGEL in this paper. The gene has one probe set in the Affymetrix Gene-Chip Human Genome U133 Plus 2.0 platform, *205891_at*.

Fig 1 shows the query interface for the web server access. The input to the system is official gene symbols, which could be looked up from the NCBI gene database (http://www.ncbi.nlm.nih.gov/gene). All probe sets representing the queried gene in the Affymetrix Gene-Chip Human Genome U133 Plus 2.0 Array platform will be shown. The gene expression patterns of each probe set are reported in one PDF file. The file includes graphs that show gene expression patterns of smokers and nonsmokers in different cells, sex difference in response to cigarette smoking, gene expression levels change with age, and the relationships between the gene expression and smoking intake. SEGEL provides some user-friendly functions to query the server. Users can enter a list of multiple gene symbols separated by spaces for the batch searching (for example, “PPARG HSPA6 ADORA2B”). Results of all genes in the list will be shown. Users can also enter a query string for the fuzzy searching (For example, “ADOR” or “PAR”). Any gene symbols containing the query string will be displayed.

The gene ADORA2B, adenosine A_2B_ receptor was used as an example to query the SEGEL. ADORA2B encodes the adenosine receptor A_2B_, a member of the G protein-coupled receptors that stimulate adenylate cyclase activity in the presence of adenosine. The ADORA2B is generally defined as “the low affinity” adenosine receptor and requires high micromolar intracellular adenosine concentrations to be activated [15, 16]. In COPD, the lungs are constantly in a hypoxic and inflammatory environment, which causes increased intracellular concentration of adenosine [17]. Higher adenosine concentrations observed in COPD patients may increase A_2B_ receptor activation [17–21]. Evidences have suggested that ADORA2B contributes to the pathogenesis of COPD. First, the ADORA2B gene plays an established role in the inflammatory processes of the lung in smokers[12]. Secondly, the interactions among the adenosine receptors (A_1_, A_2A_, A_2B_ and A_3_) can trigger signal events that can further contributes to the expression of other genes including IL-8, IL-1beta, CCL2) all of which have been previously described as COPD-related genes [12, 22, 23]. And thirdly, the engagement of the ADORA2B has been implicated in the disease progression and tissue remodeling [24–26].

**Fig 2:**
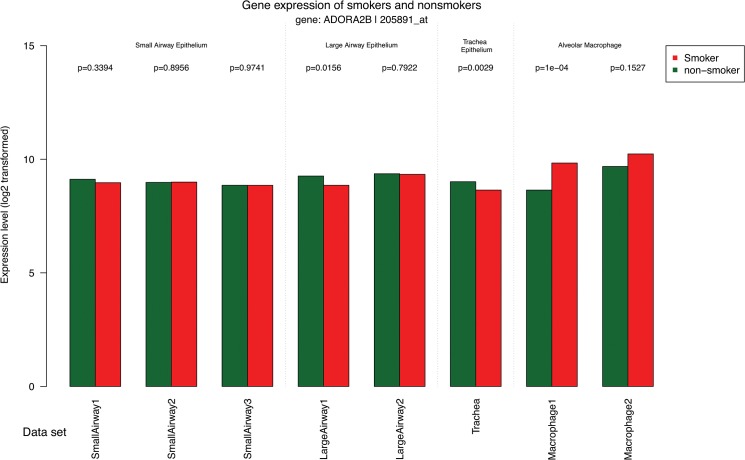
The expression levels of the gene ADORA2B in different cell types from smokers (red bars) and nonsmokers (green bars). These cell types include small airways epithelial cells, large airways epithelial cells, trachea epithelial cells, and alveolar macrophage.

**Fig 3:**
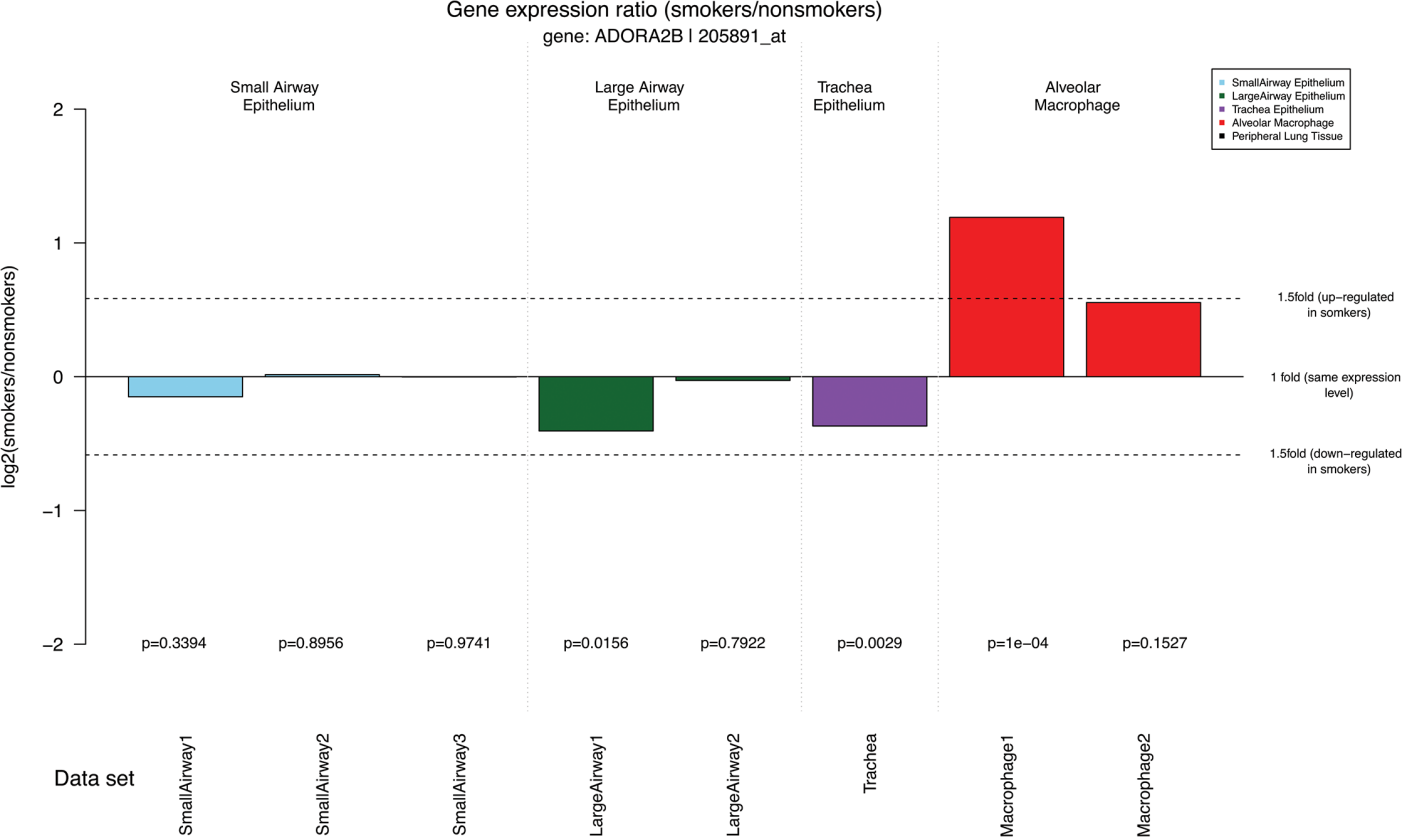
The ratio (log2-transformed) of ADORA2B expression levels in smokers vs nonsmokers. Positive and negative values in the graph demonstrated up and down regulation of the gene in smokers. The dotted line represents 1,5 fold change.

**Fig 4:**
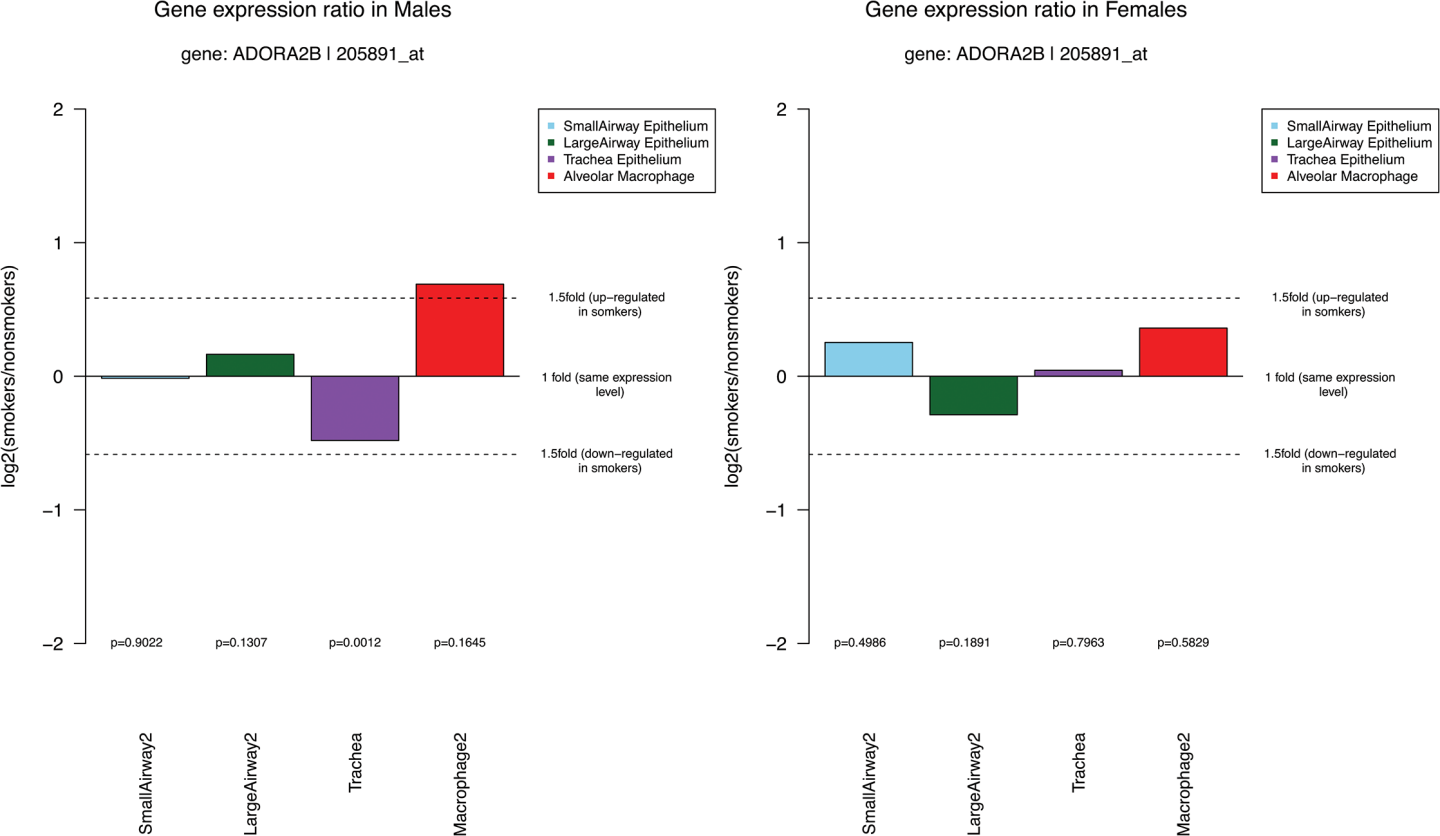
The ratio (log2-transformed) of ADORA2B expression levels in (a) male smokers *vs* male nonsmokers (b) female smokers *vs* female nonsmokers. Positive and negative values in the graph demonstrated up and down regulation of the gene in smokers. The dotted line represents 1.5 fold change.

**Fig 5:**
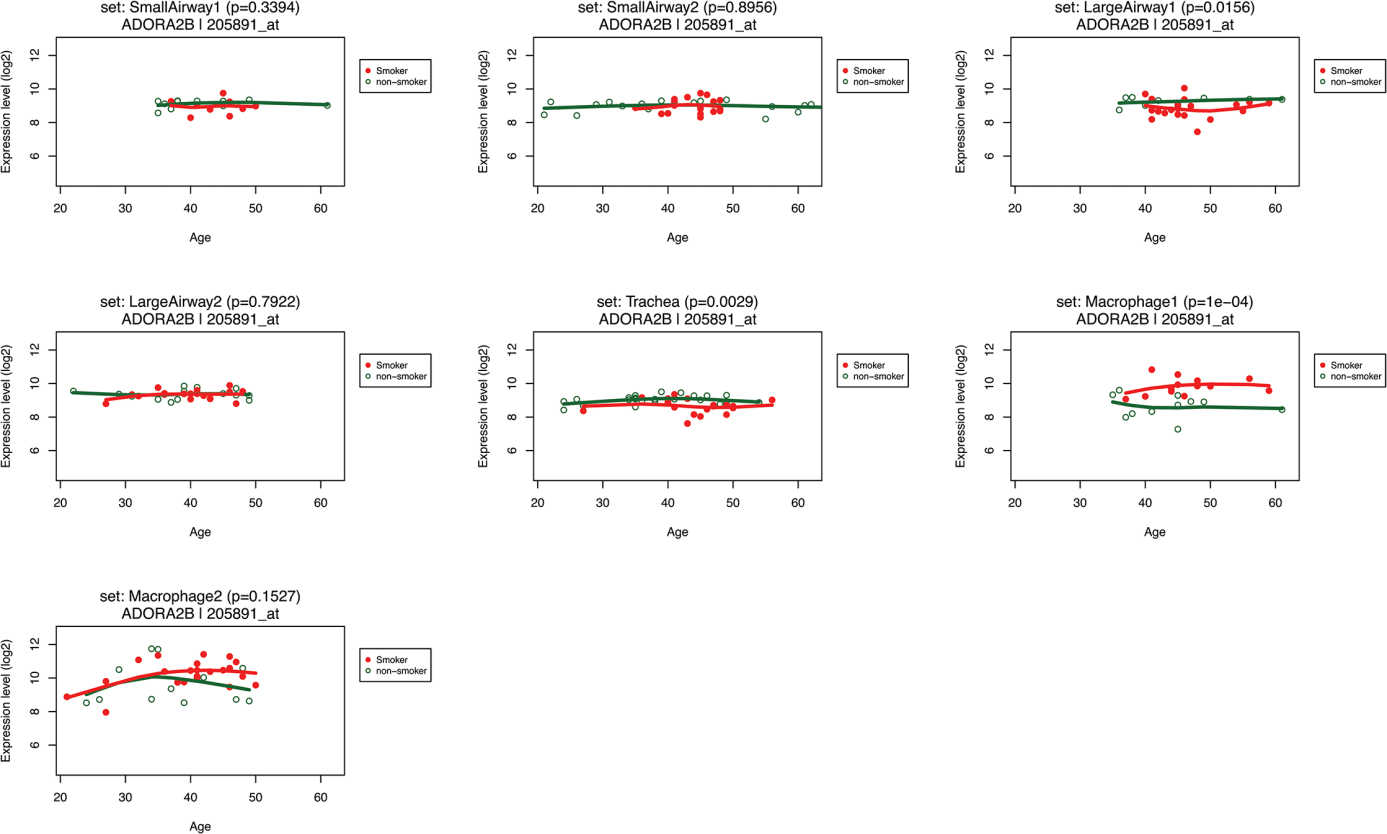
The ADORA2B expression level changes along with age in smokers (red) and nonsmokers (green).

ADORA2B has one gene probe set in the Affymetrix Gene-Chip Human Genome U133 Plus 2.0 Array platform, *205891_at*. The output results of this probe set are shown in Figs 2–5. The first graph of the output PDF file (Fig 2) shows the spatial differences of ADORA2B gene expression in small airway epithelial cells, large airway epithelial cells, trachea epithelial cells, and alveolar macrophage from smokers (red bars) and nonsmokers (green bars). The ADORA2B gene is highly expressed in alveolar macrophage from smokers as compared to epithelial cells in small airways, large airways, and trachea. Furthermore, our result demonstrated this increase expression of ADORA2B was more prominent in alveolar macrophages from smokers compared to nonsmokers. On the contrary, there were no obvious differences between smokers and nonsmokers in other types of cell To clearly display the differences of ADORA2B gene expression between smokers and nonsmokers, we calculated the gene expression ratio (log2-transformed) of smokers *vs* nonsmokers and presented the values in the second graph of the output PDF file (Fig 3). Positive and negative values in the graph demonstrated up and down regulation of genes in smokers. The dotted line represents a 1.5 fold change in gene expression. Consistent with Fig 2, ADORA2B shows a significant difference in gene expression between smokers and nonsmokers in the first data set of alveolar macrophage. Gene expression differences in other cell types were lower than a 1.5 fold. Our data suggest that ADORA2B is up-regulated in the alveolar macrophage from smokers. Alveolar macrophages are the predominant defense cells in normal lung and during conditions associated with chronic inflammation i.e., COPD [27]. Recent studies revealed stimulation of ADORA2B promotes alternative macrophage activation, which have been implicated to contributing to airway remodeling and lung dysfunction [28, 29]. Results from SEGEL further substantiate these earlier findings.

Previous studies have showed that airway diseases including COPD are influenced by sex [30]. The third graph in the output PDF file (Fig 4) shows the sex differences in response to cigarette smoke among four microarray data sets from small airway epithelial cells, large airway epithelial cells, trachea epithelial cells, and alveolar macrophage. The other four data sets were not included because the sex information was lacking or there were not enough samples. As shown in the figure, ADORA2B was significantly down regulated in trachea epithelial cells in males but the difference is less than a 1.5 fold. We observed no significant differences in either females or males in the other cell types. The gene expression of males and females in different cells shown in the figure may provide the basis for understanding the molecular mechanisms underlying the sex differences in the prevalence and severity of some smoking-related lung diseases.

There is a close relationship between aging and chronic lung diseases, particularly COPD. COPD is known as a chronic inflammatory disease of the lung that progresses slowly and the majority of the patients are elderly. The classic epidemiology studies suggest death and disability from COPD is related to the rapid decline in lung function with time [31]. We employed SEGEL to track the gene expression change along with the age. The fourth graph in the output PDF file of the web server (Fig 5) demonstrates gene expression level in smokers/nonsmokers along with their age. Seven microarray data sets with the age information were included in this plot. As shown in the figure, Y-axis and X-axis represent the gene expression level (log2-transformed) and age respectively. Alveolar macrophage shows the greatest difference in ADORA2B expression between smokers (red lines) and nonsmokers (green lines), especially after 40 years old. ADORA2B showed a general increase in smokers in alveolar macrophage from 20 to 40 years old but showed constant expression in other types of cells from both smokers and nonsmokers. SEGEL can provide evidence in gene expression levels for numerous studies [32–34] that there is an association between age and smoking-related diseases.

**Fig 6:**
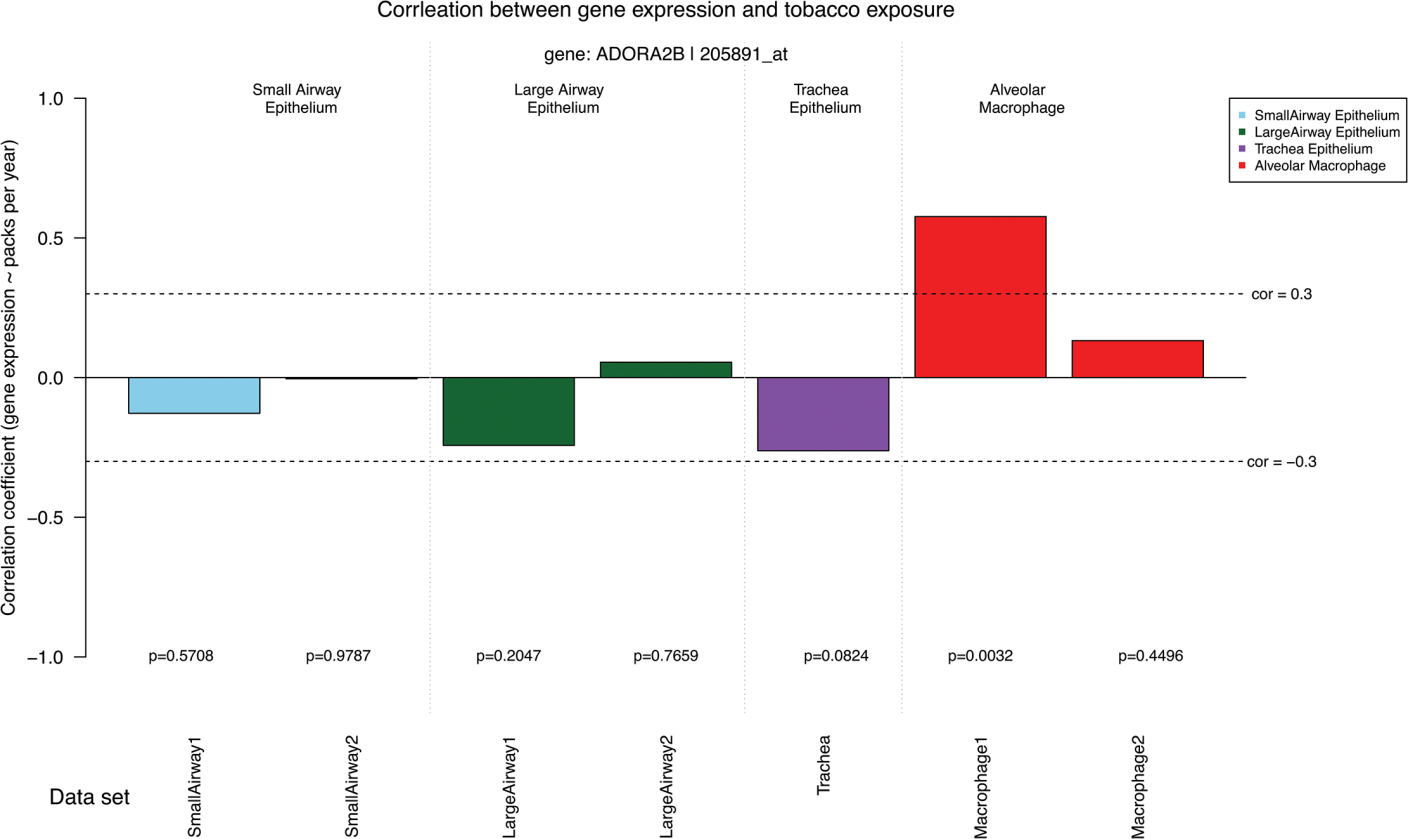
The Pearson’s correlation coefficients between ADORA2B expression levels and smoking intake (packs per year).

As we all know, heavy smokers have a higher risk than light smokers in the smoking-related diseases. The cause of death in patients with 30 or more packs per year showed significant differences compared to patients with less than 30 packs per year. Smoking pack-years is an important clinical factor in evaluating the risk of lung diseases. However, very little is known if the number of smoking pack-years is related to the gene expression levels. In the fifth graph of the output PDF file (Fig 6), the smoking intake (packs per year) was considered in the analysis. The graph provides a quantitative measurement of the effects of smoking intake on lung gene expression by calculating the Pearson’s correlation coefficients (and corresponding p-values) between gene expression levels and tobacco intake. As shown in Fig 6, the gene ADORA2B expression shows a strong positive correlation with smoking exposure in the first data set of alveolar macrophage (correlation coefficient is greater than 0.5 and p-value = 0.0032). Our data suggest that greater smoking intake will increase the expression level of ADORA2B in the alveolar macrophage from older smokers.

SEGEL is an important resource for cigarette smoking research. The web server is comprehensive in sample size, cell types and the number of genes. Previous transcriptome studies of the effects of smoking on lungs have used relatively small numbers of samples or only focused on one lung tissue or on specific genes. SEGEL combined more than 360 samples from eight public microarray data in four cell types (alveolar macrophage, small airways epithelial cells, large airways epithelial cells, and trachea epithelial cells). We also did a comprehensive analysis of more than 42,000 gene probe sets. The analysis includes the spatial difference among tissues/cells, temporal difference, sex difference in response of smoking, and the correlation between gene expression and smoking intake. It will provide deeper insights into the transcriptional foundations of smoking effects on the lungs. SEGEL will also provide useful information regarding the molecular mechanism of smoking-related lung diseases such as COPD, and aid in discovery of novel drugs. Researchers can freely access our server to check the gene expression profiles of disease-related genes, or identify which genes in which types of cells would be important for disease development.

We will expand our web server by adding more public microarray data sets in the future. All samples in our current server were profiled with Affymetrix HG-U133 Plus 2.0 GeneChip microarrays. In the next stage, we will include more samples from different platforms such as Affymetrix HG-U133A or Affymetrix Exon ST 1.0 Array. We believe information provided by the SEGEL web server on smoking effects on the lung will be more reliable with a larger sample size.

## Supporting Information

S1 Fig A boxplot of the signal intensity distributions of 362 samples from eight microarray datasets. (PDF)

S1 Table Detail information of these 362 samples integrated in the SEGEL web server. (DOCX)
