# Supplementary figures and images for: SEGEL: A Web Server for Visualization of Smoking Effects on Human Lung Gene Expression
Source: PLoS One. 2015 May 26;10(5):e0128326. doi: 10.1371/journal.pone.0128326 (PMC4444269; doi:10.1371/journal.pone.0128326)

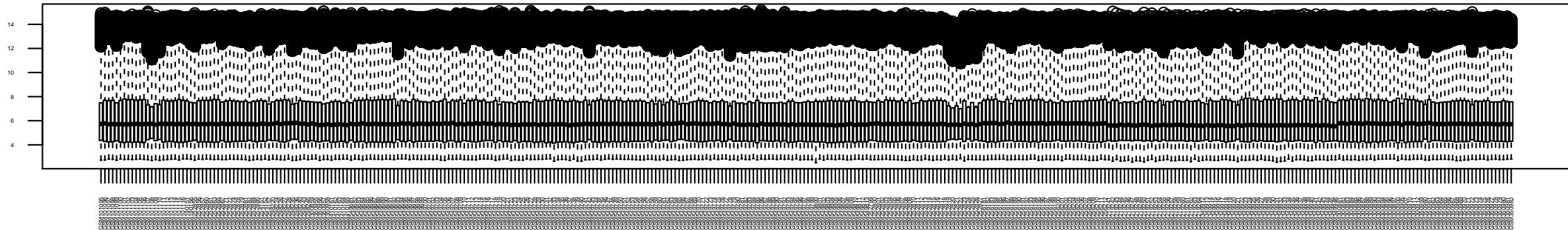

Supplement: S1 Fig — (PDF) [file pone.0128326.s001.pdf]
