# Supplementary material for: SEGEL: A Web Server for Visualization of Smoking Effects on Human Lung Gene Expression
Source: PLoS One. 2015 May 26;10(5):e0128326. doi: 10.1371/journal.pone.0128326 (PMC4444269; doi:10.1371/journal.pone.0128326)
Supplement: S1 Table — (DOCX) [file pone.0128326.s002.docx]

| GEO Sample Entry | Age | Gender | Smoking Status | Pack-year | Cell/Tissue | Data Set Name | GEO Data Set |
| --- | --- | --- | --- | --- | --- | --- | --- |
| GSM927630 | NA | NA | 0 | NA | Peripheral Lung | Peripheral Lung | GSE37768 |
| GSM927631 | NA | NA | 0 | NA | Peripheral Lung | Peripheral Lung | GSE37768 |
| GSM927632 | NA | NA | 0 | NA | Peripheral Lung | Peripheral Lung | GSE37768 |
| GSM927633 | NA | NA | 0 | NA | Peripheral Lung | Peripheral Lung | GSE37768 |
| GSM927634 | NA | NA | 0 | NA | Peripheral Lung | Peripheral Lung | GSE37768 |
| GSM927635 | NA | NA | 0 | NA | Peripheral Lung | Peripheral Lung | GSE37768 |
| GSM927636 | NA | NA | 0 | NA | Peripheral Lung | Peripheral Lung | GSE37768 |
| GSM927637 | NA | NA | 0 | NA | Peripheral Lung | Peripheral Lung | GSE37768 |
| GSM927638 | NA | NA | 0 | NA | Peripheral Lung | Peripheral Lung | GSE37768 |
| GSM927639 | NA | NA | 1 | NA | Peripheral Lung | Peripheral Lung | GSE37768 |
| GSM927640 | NA | NA | 1 | NA | Peripheral Lung | Peripheral Lung | GSE37768 |
| GSM927641 | NA | NA | 1 | NA | Peripheral Lung | Peripheral Lung | GSE37768 |
| GSM927642 | NA | NA | 1 | NA | Peripheral Lung | Peripheral Lung | GSE37768 |
| GSM927643 | NA | NA | 1 | NA | Peripheral Lung | Peripheral Lung | GSE37768 |
| GSM927644 | NA | NA | 1 | NA | Peripheral Lung | Peripheral Lung | GSE37768 |
| GSM927645 | NA | NA | 1 | NA | Peripheral Lung | Peripheral Lung | GSE37768 |
| GSM927646 | NA | NA | 1 | NA | Peripheral Lung | Peripheral Lung | GSE37768 |
| GSM927647 | NA | NA | 1 | NA | Peripheral Lung | Peripheral Lung | GSE37768 |
| GSM927648 | NA | NA | 1 | NA | Peripheral Lung | Peripheral Lung | GSE37768 |
| GSM927649 | NA | NA | 1 | NA | Peripheral Lung | Peripheral Lung | GSE37768 |
| GSM300881 | 35 | M | 0 | 0 | Treachea | Treachea | GSE13933 |
| GSM300882 | 35 | M | 0 | 0 | Treachea | Treachea | GSE13933 |
| GSM300883 | 49 | M | 0 | 0 | Treachea | Treachea | GSE13933 |
| GSM300884 | 38 | M | 0 | 0 | Treachea | Treachea | GSE13933 |
| GSM300885 | 38 | M | 0 | 0 | Treachea | Treachea | GSE13933 |
| GSM300886 | 46 | M | 0 | 0 | Treachea | Treachea | GSE13933 |
| GSM300887 | 49 | F | 0 | 0 | Treachea | Treachea | GSE13933 |
| GSM300888 | 34 | M | 0 | 0 | Treachea | Treachea | GSE13933 |
| GSM300889 | 45 | F | 0 | 0 | Treachea | Treachea | GSE13933 |
| GSM300890 | 44 | M | 0 | 0 | Treachea | Treachea | GSE13933 |
| GSM300893 | 49 | M | 0 | 0 | Treachea | Treachea | GSE13933 |
| GSM300894 | 36 | F | 0 | 0 | Treachea | Treachea | GSE13933 |
| GSM300895 | 38 | M | 0 | 0 | Treachea | Treachea | GSE13933 |
| GSM300896 | 35 | M | 0 | 0 | Treachea | Treachea | GSE13933 |
| GSM300897 | 39 | M | 0 | 0 | Treachea | Treachea | GSE13933 |
| GSM300900 | 56 | M | 1 | 80 | Treachea | Treachea | GSE13933 |
| GSM300905 | 43 | M | 1 | 30 | Treachea | Treachea | GSE13933 |
| GSM300907 | 41 | M | 1 | 12 | Treachea | Treachea | GSE13933 |
| GSM300910 | 49 | M | 1 | 16 | Treachea | Treachea | GSE13933 |
| GSM300911 | 40 | M | 1 | 44.3 | Treachea | Treachea | GSE13933 |
| GSM300912 | 45 | M | 1 | 23.5 | Treachea | Treachea | GSE13933 |
| GSM350959 | 42 | M | 0 | 0 | Treachea | Treachea | GSE13933 |
| GSM350960 | 41 | F | 0 | 0 | Treachea | Treachea | GSE13933 |
| GSM350961 | 34 | F | 0 | 0 | Treachea | Treachea | GSE13933 |
| GSM350962 | 26 | M | 0 | 0 | Treachea | Treachea | GSE13933 |
| GSM350963 | 54 | F | 0 | 0 | Treachea | Treachea | GSE13933 |
| GSM350964 | 24 | M | 0 | 0 | Treachea | Treachea | GSE13933 |
| GSM350965 | 49 | M | 0 | 0 | Treachea | Treachea | GSE13933 |
| GSM350966 | 27 | F | 0 | 0 | Treachea | Treachea | GSE13933 |
| GSM350967 | 48 | F | 0 | 0 | Treachea | Treachea | GSE13933 |
| GSM350968 | 35 | M | 0 | 0 | Treachea | Treachea | GSE13933 |
| GSM350969 | 24 | F | 0 | 0 | Treachea | Treachea | GSE13933 |
| GSM350970 | 50 | F | 1 | 22 | Treachea | Treachea | GSE13933 |
| GSM350971 | 40 | F | 1 | 47 | Treachea | Treachea | GSE13933 |
| GSM350972 | 47 | M | 1 | 33 | Treachea | Treachea | GSE13933 |
| GSM350973 | 41 | M | 1 | 38 | Treachea | Treachea | GSE13933 |
| GSM350974 | 40 | M | 1 | 40 | Treachea | Treachea | GSE13933 |
| GSM350975 | 43 | F | 1 | 17.5 | Treachea | Treachea | GSE13933 |
| GSM350976 | 36 | M | 1 | 24.5 | Treachea | Treachea | GSE13933 |
| GSM350977 | 44 | M | 1 | 10 | Treachea | Treachea | GSE13933 |
| GSM350978 | 46 | M | 1 | 26 | Treachea | Treachea | GSE13933 |
| GSM350979 | 47 | M | 1 | 24 | Treachea | Treachea | GSE13933 |
| GSM350980 | 27 | M | 1 | 9.25 | Treachea | Treachea | GSE13933 |
| GSM350981 | 50 | M | 1 | 16.4 | Treachea | Treachea | GSE13933 |
| GSM350982 | 49 | M | 1 | 22 | Treachea | Treachea | GSE13933 |
| GSM252799 | 61 | M | 0 | 0 | LargeAirway | LargeAirway1 | GSE10006 |
| GSM252800 | 37 | M | 0 | 0 | LargeAirway | LargeAirway1 | GSE10006 |
| GSM252801 | 38 | M | 0 | 0 | LargeAirway | LargeAirway1 | GSE10006 |
| GSM252802 | 36 | M | 0 | 0 | LargeAirway | LargeAirway1 | GSE10006 |
| GSM252803 | 49 | M | 0 | 0 | LargeAirway | LargeAirway1 | GSE10006 |
| GSM252804 | 40 | M | 0 | 0 | LargeAirway | LargeAirway1 | GSE10006 |
| GSM252805 | 45 | M | 0 | 0 | LargeAirway | LargeAirway1 | GSE10006 |
| GSM252806 | 42 | M | 0 | 0 | LargeAirway | LargeAirway1 | GSE10006 |
| GSM252807 | 56 | M | 0 | 0 | LargeAirway | LargeAirway1 | GSE10006 |
| GSM252808 | 46 | M | 1 | 21 | LargeAirway | LargeAirway1 | GSE10006 |
| GSM252809 | 40 | F | 1 | 25 | LargeAirway | LargeAirway1 | GSE10006 |
| GSM252810 | 42 | M | 1 | 20 | LargeAirway | LargeAirway1 | GSE10006 |
| GSM252811 | 44 | F | 1 | 30 | LargeAirway | LargeAirway1 | GSE10006 |
| GSM252812 | 46 | F | 1 | 23 | LargeAirway | LargeAirway1 | GSE10006 |
| GSM252813 | 45 | M | 1 | 24 | LargeAirway | LargeAirway1 | GSE10006 |
| GSM252814 | 56 | M | 1 | 80 | LargeAirway | LargeAirway1 | GSE10006 |
| GSM252815 | 59 | M | 1 | 60 | LargeAirway | LargeAirway1 | GSE10006 |
| GSM252816 | 45 | M | 1 | 70 | LargeAirway | LargeAirway1 | GSE10006 |
| GSM252817 | 41 | M | 1 | 20 | LargeAirway | LargeAirway1 | GSE10006 |
| GSM252818 | 47 | M | 1 | 29 | LargeAirway | LargeAirway1 | GSE10006 |
| GSM252819 | 41 | M | 1 | 45 | LargeAirway | LargeAirway1 | GSE10006 |
| GSM252820 | 48 | M | 1 | 32 | LargeAirway | LargeAirway1 | GSE10006 |
| GSM252821 | 55 | F | 1 | 70 | LargeAirway | LargeAirway1 | GSE10006 |
| GSM252822 | 43 | F | 1 | 36 | LargeAirway | LargeAirway1 | GSE10006 |
| GSM252823 | 54 | F | 1 | 45 | LargeAirway | LargeAirway1 | GSE10006 |
| GSM252824 | 41 | M | 1 | 15 | LargeAirway | LargeAirway1 | GSE10006 |
| GSM252825 | 45 | M | 1 | 23 | LargeAirway | LargeAirway1 | GSE10006 |
| GSM252826 | 50 | F | 1 | 22 | LargeAirway | LargeAirway1 | GSE10006 |
| GSM252827 | 46 | F | 1 | 33 | LargeAirway | LargeAirway1 | GSE10006 |
| GSM256181 | 45 | F | 0 | 0 | LargeAirway | LargeAirway2 | GSE10135 |
| GSM256182 | 47 | F | 0 | 0 | LargeAirway | LargeAirway2 | GSE10135 |
| GSM256183 | 29 | F | 0 | 0 | LargeAirway | LargeAirway2 | GSE10135 |
| GSM256184 | 39 | F | 0 | 0 | LargeAirway | LargeAirway2 | GSE10135 |
| GSM256185 | 38 | M | 0 | 0 | LargeAirway | LargeAirway2 | GSE10135 |
| GSM256186 | 49 | M | 0 | 1 | LargeAirway | LargeAirway2 | GSE10135 |
| GSM256187 | 41 | F | 0 | 0 | LargeAirway | LargeAirway2 | GSE10135 |
| GSM256188 | 47 | M | 0 | 0 | LargeAirway | LargeAirway2 | GSE10135 |
| GSM256189 | 49 | M | 0 | 0 | LargeAirway | LargeAirway2 | GSE10135 |
| GSM256190 | 39 | M | 0 | 0 | LargeAirway | LargeAirway2 | GSE10135 |
| GSM256191 | 35 | M | 0 | 0 | LargeAirway | LargeAirway2 | GSE10135 |
| GSM256192 | 22 | F | 0 | 0 | LargeAirway | LargeAirway2 | GSE10135 |
| GSM256193 | 31 | M | 0 | 0 | LargeAirway | LargeAirway2 | GSE10135 |
| GSM256194 | 37 | M | 0 | 0 | LargeAirway | LargeAirway2 | GSE10135 |
| GSM256195 | 39 | M | 1 | 30 | LargeAirway | LargeAirway2 | GSE10135 |
| GSM256196 | 36 | M | 1 | 3 | LargeAirway | LargeAirway2 | GSE10135 |
| GSM256197 | 43 | M | 1 | 30 | LargeAirway | LargeAirway2 | GSE10135 |
| GSM256198 | 46 | F | 1 | 19 | LargeAirway | LargeAirway2 | GSE10135 |
| GSM256199 | 47 | M | 1 | 11 | LargeAirway | LargeAirway2 | GSE10135 |
| GSM256200 | 43 | M | 1 | 10 | LargeAirway | LargeAirway2 | GSE10135 |
| GSM256201 | 41 | M | 1 | 12 | LargeAirway | LargeAirway2 | GSE10135 |
| GSM256202 | 42 | F | 1 | 20 | LargeAirway | LargeAirway2 | GSE10135 |
| GSM256203 | 36 | M | 1 | 11.5 | LargeAirway | LargeAirway2 | GSE10135 |
| GSM256204 | 46 | M | 1 | 26 | LargeAirway | LargeAirway2 | GSE10135 |
| GSM256205 | 41 | M | 1 | 13 | LargeAirway | LargeAirway2 | GSE10135 |
| GSM256206 | 40 | M | 1 | 24 | LargeAirway | LargeAirway2 | GSE10135 |
| GSM256207 | 31 | M | 1 | 12.5 | LargeAirway | LargeAirway2 | GSE10135 |
| GSM256208 | 27 | F | 1 | 3.8 | LargeAirway | LargeAirway2 | GSE10135 |
| GSM256209 | 40 | F | 1 | 47 | LargeAirway | LargeAirway2 | GSE10135 |
| GSM256210 | 32 | F | 1 | 7.6 | LargeAirway | LargeAirway2 | GSE10135 |
| GSM256211 | 48 | M | 1 | 43 | LargeAirway | LargeAirway2 | GSE10135 |
| GSM256212 | 35 | M | 1 | 5 | LargeAirway | LargeAirway2 | GSE10135 |
| GSM101095 | 41 | M | 0 | 0 | SmallAirway | SmallAirway1 | GSE4498 |
| GSM101096 | 35 | M | 0 | 0 | SmallAirway | SmallAirway1 | GSE4498 |
| GSM101097 | 61 | M | 0 | 0 | SmallAirway | SmallAirway1 | GSE4498 |
| GSM101098 | 37 | F | 0 | 0 | SmallAirway | SmallAirway1 | GSE4498 |
| GSM101099 | 45 | M | 0 | 0 | SmallAirway | SmallAirway1 | GSE4498 |
| GSM101100 | 47 | M | 0 | 0 | SmallAirway | SmallAirway1 | GSE4498 |
| GSM101101 | 38 | M | 0 | 0 | SmallAirway | SmallAirway1 | GSE4498 |
| GSM101102 | 49 | F | 0 | 0 | SmallAirway | SmallAirway1 | GSE4498 |
| GSM101103 | 45 | M | 0 | 0 | SmallAirway | SmallAirway1 | GSE4498 |
| GSM101104 | 36 | M | 0 | 0 | SmallAirway | SmallAirway1 | GSE4498 |
| GSM101105 | 38 | M | 0 | 0 | SmallAirway | SmallAirway1 | GSE4498 |
| GSM101106 | 35 | M | 0 | 0 | SmallAirway | SmallAirway1 | GSE4498 |
| GSM101107 | 46 | M | 1 | 21 | SmallAirway | SmallAirway1 | GSE4498 |
| GSM101108 | 40 | F | 1 | 25 | SmallAirway | SmallAirway1 | GSE4498 |
| GSM101109 | 44 | M | 1 | 45 | SmallAirway | SmallAirway1 | GSE4498 |
| GSM101110 | 43 | M | 1 | 15 | SmallAirway | SmallAirway1 | GSE4498 |
| GSM101111 | 37 | F | 1 | 23 | SmallAirway | SmallAirway1 | GSE4498 |
| GSM101112 | 41 | M | 1 | 20 | SmallAirway | SmallAirway1 | GSE4498 |
| GSM101113 | 45 | M | 1 | 28 | SmallAirway | SmallAirway1 | GSE4498 |
| GSM101114 | 48 | M | 1 | 20 | SmallAirway | SmallAirway1 | GSE4498 |
| GSM101115 | 50 | M | 1 | 38 | SmallAirway | SmallAirway1 | GSE4498 |
| GSM101116 | 46 | F | 1 | 23 | SmallAirway | SmallAirway1 | GSE4498 |
| GSM190151 | 44 | M | 0 | 0 | SmallAirway | SmallAirway2 | GSE13933 |
| GSM190156 | 56 | M | 0 | 0 | SmallAirway | SmallAirway2 | GSE13933 |
| GSM252855 | 41 | M | 0 | 0 | SmallAirway | SmallAirway2 | GSE13933 |
| GSM252856 | 61 | M | 0 | 0 | SmallAirway | SmallAirway2 | GSE13933 |
| GSM252857 | 37 | F | 0 | 0 | SmallAirway | SmallAirway2 | GSE13933 |
| GSM252860 | 47 | M | 0 | 0 | SmallAirway | SmallAirway2 | GSE13933 |
| GSM252861 | 36 | M | 0 | 0 | SmallAirway | SmallAirway2 | GSE13933 |
| GSM252863 | 45 | M | 0 | 0 | SmallAirway | SmallAirway2 | GSE13933 |
| GSM252865 | 49 | F | 0 | 0 | SmallAirway | SmallAirway2 | GSE13933 |
| GSM252867 | 45 | M | 0 | 0 | SmallAirway | SmallAirway2 | GSE13933 |
| GSM252871 | 40 | M | 1 | 24 | SmallAirway | SmallAirway2 | GSE13933 |
| GSM252872 | 48 | M | 1 | 20 | SmallAirway | SmallAirway2 | GSE13933 |
| GSM252874 | 45 | M | 1 | 37 | SmallAirway | SmallAirway2 | GSE13933 |
| GSM252876 | 45 | M | 1 | 24 | SmallAirway | SmallAirway2 | GSE13933 |
| GSM252879 | 41 | M | 1 | 20 | SmallAirway | SmallAirway2 | GSE13933 |
| GSM252881 | 41 | M | 1 | 45 | SmallAirway | SmallAirway2 | GSE13933 |
| GSM252882 | 48 | M | 1 | 32 | SmallAirway | SmallAirway2 | GSE13933 |
| GSM252884 | 43 | F | 1 | 36 | SmallAirway | SmallAirway2 | GSE13933 |
| GSM252885 | 41 | M | 1 | 15 | SmallAirway | SmallAirway2 | GSE13933 |
| GSM254151 | 37 | M | 0 | 0 | SmallAirway | SmallAirway2 | GSE13933 |
| GSM254152 | 31 | M | 0 | 0 | SmallAirway | SmallAirway2 | GSE13933 |
| GSM254157 | 45 | M | 1 | 23 | SmallAirway | SmallAirway2 | GSE13933 |
| GSM298220 | 60 | M | 0 | 0 | SmallAirway | SmallAirway2 | GSE13933 |
| GSM298224 | 73 | M | 0 | 0 | SmallAirway | SmallAirway2 | GSE13933 |
| GSM298226 | 22 | F | 0 | 0 | SmallAirway | SmallAirway2 | GSE13933 |
| GSM298227 | 29 | M | 0 | 0 | SmallAirway | SmallAirway2 | GSE13933 |
| GSM298228 | 39 | F | 0 | 0 | SmallAirway | SmallAirway2 | GSE13933 |
| GSM298235 | 46 | F | 1 | 19 | SmallAirway | SmallAirway2 | GSE13933 |
| GSM298236 | 47 | M | 1 | 11 | SmallAirway | SmallAirway2 | GSE13933 |
| GSM298240 | 41 | M | 1 | 13 | SmallAirway | SmallAirway2 | GSE13933 |
| GSM298243 | 35 | M | 1 | 5 | SmallAirway | SmallAirway2 | GSE13933 |
| GSM298245 | 48 | M | 1 | 43 | SmallAirway | SmallAirway2 | GSE13933 |
| GSM300859 | 62 | F | 0 | 0 | SmallAirway | SmallAirway2 | GSE13933 |
| GSM350955 | 26 | M | 0 | 0 | SmallAirway | SmallAirway2 | GSE13933 |
| GSM350956 | 33 | F | 0 | 0 | SmallAirway | SmallAirway2 | GSE13933 |
| GSM350957 | 45 | M | 1 | 46 | SmallAirway | SmallAirway2 | GSE13933 |
| GSM350958 | 48 | F | 1 | 26.5 | SmallAirway | SmallAirway2 | GSE13933 |
| GSM410161 | 21 | F | 0 | 0 | SmallAirway | SmallAirway2 | GSE13933 |
| GSM410162 | 45 | M | 0 | 0 | SmallAirway | SmallAirway2 | GSE13933 |
| GSM410163 | 55 | M | 0 | 0 | SmallAirway | SmallAirway2 | GSE13933 |
| GSM410164 | 47 | M | 1 | 45 | SmallAirway | SmallAirway2 | GSE13933 |
| GSM410165 | 39 | M | 1 | 11 | SmallAirway | SmallAirway2 | GSE13933 |
| GSM549681 | NA* | NA | 0 | NA | SmallAirway | SmallAirway3 | GSE30063 |
| GSM549682 | NA | NA | 0 | NA | SmallAirway | SmallAirway3 | GSE30063 |
| GSM549683 | NA | NA | 0 | NA | SmallAirway | SmallAirway3 | GSE30063 |
| GSM549684 | NA | NA | 0 | NA | SmallAirway | SmallAirway3 | GSE30063 |
| GSM549685 | NA | NA | 0 | NA | SmallAirway | SmallAirway3 | GSE30063 |
| GSM549686 | NA | NA | 0 | NA | SmallAirway | SmallAirway3 | GSE30063 |
| GSM549687 | NA | NA | 0 | NA | SmallAirway | SmallAirway3 | GSE30063 |
| GSM549688 | NA | NA | 0 | NA | SmallAirway | SmallAirway3 | GSE30063 |
| GSM549689 | NA | NA | 0 | NA | SmallAirway | SmallAirway3 | GSE30063 |
| GSM549690 | NA | NA | 0 | NA | SmallAirway | SmallAirway3 | GSE30063 |
| GSM549691 | NA | NA | 0 | NA | SmallAirway | SmallAirway3 | GSE30063 |
| GSM549692 | NA | NA | 0 | NA | SmallAirway | SmallAirway3 | GSE30063 |
| GSM549693 | NA | NA | 0 | NA | SmallAirway | SmallAirway3 | GSE30063 |
| GSM549694 | NA | NA | 0 | NA | SmallAirway | SmallAirway3 | GSE30063 |
| GSM549695 | NA | NA | 0 | NA | SmallAirway | SmallAirway3 | GSE30063 |
| GSM549696 | NA | NA | 0 | NA | SmallAirway | SmallAirway3 | GSE30063 |
| GSM549697 | NA | NA | 0 | NA | SmallAirway | SmallAirway3 | GSE30063 |
| GSM549698 | NA | NA | 0 | NA | SmallAirway | SmallAirway3 | GSE30063 |
| GSM549699 | NA | NA | 0 | NA | SmallAirway | SmallAirway3 | GSE30063 |
| GSM549700 | NA | NA | 0 | NA | SmallAirway | SmallAirway3 | GSE30063 |
| GSM549701 | NA | NA | 0 | NA | SmallAirway | SmallAirway3 | GSE30063 |
| GSM549702 | NA | NA | 0 | NA | SmallAirway | SmallAirway3 | GSE30063 |
| GSM549703 | NA | NA | 0 | NA | SmallAirway | SmallAirway3 | GSE30063 |
| GSM549704 | NA | NA | 0 | NA | SmallAirway | SmallAirway3 | GSE30063 |
| GSM549705 | NA | NA | 0 | NA | SmallAirway | SmallAirway3 | GSE30063 |
| GSM549706 | NA | NA | 0 | NA | SmallAirway | SmallAirway3 | GSE30063 |
| GSM549707 | NA | NA | 0 | NA | SmallAirway | SmallAirway3 | GSE30063 |
| GSM549708 | NA | NA | 0 | NA | SmallAirway | SmallAirway3 | GSE30063 |
| GSM549709 | NA | NA | 0 | NA | SmallAirway | SmallAirway3 | GSE30063 |
| GSM549710 | NA | NA | 0 | NA | SmallAirway | SmallAirway3 | GSE30063 |
| GSM549711 | NA | NA | 0 | NA | SmallAirway | SmallAirway3 | GSE30063 |
| GSM549712 | NA | NA | 0 | NA | SmallAirway | SmallAirway3 | GSE30063 |
| GSM549713 | NA | NA | 0 | NA | SmallAirway | SmallAirway3 | GSE30063 |
| GSM549714 | NA | NA | 0 | NA | SmallAirway | SmallAirway3 | GSE30063 |
| GSM549715 | NA | NA | 0 | NA | SmallAirway | SmallAirway3 | GSE30063 |
| GSM549716 | NA | NA | 0 | NA | SmallAirway | SmallAirway3 | GSE30063 |
| GSM549717 | NA | NA | 0 | NA | SmallAirway | SmallAirway3 | GSE30063 |
| GSM549718 | NA | NA | 0 | NA | SmallAirway | SmallAirway3 | GSE30063 |
| GSM549719 | NA | NA | 0 | NA | SmallAirway | SmallAirway3 | GSE30063 |
| GSM549720 | NA | NA | 0 | NA | SmallAirway | SmallAirway3 | GSE30063 |
| GSM549721 | NA | NA | 0 | NA | SmallAirway | SmallAirway3 | GSE30063 |
| GSM549722 | NA | NA | 0 | NA | SmallAirway | SmallAirway3 | GSE30063 |
| GSM549723 | NA | NA | 0 | NA | SmallAirway | SmallAirway3 | GSE30063 |
| GSM549724 | NA | NA | 0 | NA | SmallAirway | SmallAirway3 | GSE30063 |
| GSM549725 | NA | NA | 0 | NA | SmallAirway | SmallAirway3 | GSE30063 |
| GSM549726 | NA | NA | 0 | NA | SmallAirway | SmallAirway3 | GSE30063 |
| GSM549727 | NA | NA | 0 | NA | SmallAirway | SmallAirway3 | GSE30063 |
| GSM549728 | NA | NA | 0 | NA | SmallAirway | SmallAirway3 | GSE30063 |
| GSM549729 | NA | NA | 0 | NA | SmallAirway | SmallAirway3 | GSE30063 |
| GSM549730 | NA | NA | 0 | NA | SmallAirway | SmallAirway3 | GSE30063 |
| GSM549731 | NA | NA | 0 | NA | SmallAirway | SmallAirway3 | GSE30063 |
| GSM549732 | NA | NA | 0 | NA | SmallAirway | SmallAirway3 | GSE30063 |
| GSM549733 | NA | NA | 0 | NA | SmallAirway | SmallAirway3 | GSE30063 |
| GSM549734 | NA | NA | 0 | NA | SmallAirway | SmallAirway3 | GSE30063 |
| GSM549735 | NA | NA | 0 | NA | SmallAirway | SmallAirway3 | GSE30063 |
| GSM549736 | NA | NA | 0 | NA | SmallAirway | SmallAirway3 | GSE30063 |
| GSM549737 | NA | NA | 0 | NA | SmallAirway | SmallAirway3 | GSE30063 |
| GSM549738 | NA | NA | 0 | NA | SmallAirway | SmallAirway3 | GSE30063 |
| GSM549739 | NA | NA | 0 | NA | SmallAirway | SmallAirway3 | GSE30063 |
| GSM549740 | NA | NA | 0 | NA | SmallAirway | SmallAirway3 | GSE30063 |
| GSM549741 | NA | NA | 1 | NA | SmallAirway | SmallAirway3 | GSE30063 |
| GSM549742 | NA | NA | 1 | NA | SmallAirway | SmallAirway3 | GSE30063 |
| GSM549743 | NA | NA | 1 | NA | SmallAirway | SmallAirway3 | GSE30063 |
| GSM549744 | NA | NA | 1 | NA | SmallAirway | SmallAirway3 | GSE30063 |
| GSM549745 | NA | NA | 1 | NA | SmallAirway | SmallAirway3 | GSE30063 |
| GSM549746 | NA | NA | 1 | NA | SmallAirway | SmallAirway3 | GSE30063 |
| GSM549747 | NA | NA | 1 | NA | SmallAirway | SmallAirway3 | GSE30063 |
| GSM549748 | NA | NA | 1 | NA | SmallAirway | SmallAirway3 | GSE30063 |
| GSM549749 | NA | NA | 1 | NA | SmallAirway | SmallAirway3 | GSE30063 |
| GSM549750 | NA | NA | 1 | NA | SmallAirway | SmallAirway3 | GSE30063 |
| GSM549751 | NA | NA | 1 | NA | SmallAirway | SmallAirway3 | GSE30063 |
| GSM549752 | NA | NA | 1 | NA | SmallAirway | SmallAirway3 | GSE30063 |
| GSM549753 | NA | NA | 1 | NA | SmallAirway | SmallAirway3 | GSE30063 |
| GSM549754 | NA | NA | 1 | NA | SmallAirway | SmallAirway3 | GSE30063 |
| GSM549755 | NA | NA | 1 | NA | SmallAirway | SmallAirway3 | GSE30063 |
| GSM549756 | NA | NA | 1 | NA | SmallAirway | SmallAirway3 | GSE30063 |
| GSM549757 | NA | NA | 1 | NA | SmallAirway | SmallAirway3 | GSE30063 |
| GSM549758 | NA | NA | 1 | NA | SmallAirway | SmallAirway3 | GSE30063 |
| GSM549759 | NA | NA | 1 | NA | SmallAirway | SmallAirway3 | GSE30063 |
| GSM549760 | NA | NA | 1 | NA | SmallAirway | SmallAirway3 | GSE30063 |
| GSM549761 | NA | NA | 1 | NA | SmallAirway | SmallAirway3 | GSE30063 |
| GSM549762 | NA | NA | 1 | NA | SmallAirway | SmallAirway3 | GSE30063 |
| GSM549763 | NA | NA | 1 | NA | SmallAirway | SmallAirway3 | GSE30063 |
| GSM549764 | NA | NA | 1 | NA | SmallAirway | SmallAirway3 | GSE30063 |
| GSM549765 | NA | NA | 1 | NA | SmallAirway | SmallAirway3 | GSE30063 |
| GSM549766 | NA | NA | 1 | NA | SmallAirway | SmallAirway3 | GSE30063 |
| GSM549767 | NA | NA | 1 | NA | SmallAirway | SmallAirway3 | GSE30063 |
| GSM549768 | NA | NA | 1 | NA | SmallAirway | SmallAirway3 | GSE30063 |
| GSM549769 | NA | NA | 1 | NA | SmallAirway | SmallAirway3 | GSE30063 |
| GSM549770 | NA | NA | 1 | NA | SmallAirway | SmallAirway3 | GSE30063 |
| GSM549771 | NA | NA | 1 | NA | SmallAirway | SmallAirway3 | GSE30063 |
| GSM549772 | NA | NA | 1 | NA | SmallAirway | SmallAirway3 | GSE30063 |
| GSM549773 | NA | NA | 1 | NA | SmallAirway | SmallAirway3 | GSE30063 |
| GSM549774 | NA | NA | 1 | NA | SmallAirway | SmallAirway3 | GSE30063 |
| GSM549775 | NA | NA | 1 | NA | SmallAirway | SmallAirway3 | GSE30063 |
| GSM549776 | NA | NA | 1 | NA | SmallAirway | SmallAirway3 | GSE30063 |
| GSM549777 | NA | NA | 1 | NA | SmallAirway | SmallAirway3 | GSE30063 |
| GSM549778 | NA | NA | 1 | NA | SmallAirway | SmallAirway3 | GSE30063 |
| GSM549779 | NA | NA | 1 | NA | SmallAirway | SmallAirway3 | GSE30063 |
| GSM549780 | NA | NA | 1 | NA | SmallAirway | SmallAirway3 | GSE30063 |
| GSM549781 | NA | NA | 1 | NA | SmallAirway | SmallAirway3 | GSE30063 |
| GSM549782 | NA | NA | 1 | NA | SmallAirway | SmallAirway3 | GSE30063 |
| GSM549783 | NA | NA | 1 | NA | SmallAirway | SmallAirway3 | GSE30063 |
| GSM549784 | NA | NA | 1 | NA | SmallAirway | SmallAirway3 | GSE30063 |
| GSM549785 | NA | NA | 1 | NA | SmallAirway | SmallAirway3 | GSE30063 |
| GSM549786 | NA | NA | 1 | NA | SmallAirway | SmallAirway3 | GSE30063 |
| GSM549787 | NA | NA | 1 | NA | SmallAirway | SmallAirway3 | GSE30063 |
| GSM549788 | NA | NA | 1 | NA | SmallAirway | SmallAirway3 | GSE30063 |
| GSM549789 | NA | NA | 1 | NA | SmallAirway | SmallAirway3 | GSE30063 |
| GSM549790 | NA | NA | 1 | NA | SmallAirway | SmallAirway3 | GSE30063 |
| GSM549791 | NA | NA | 1 | NA | SmallAirway | SmallAirway3 | GSE30063 |
| GSM549792 | NA | NA | 1 | NA | SmallAirway | SmallAirway3 | GSE30063 |
| GSM549793 | NA | NA | 1 | NA | SmallAirway | SmallAirway3 | GSE30063 |
| GSM549794 | NA | NA | 1 | NA | SmallAirway | SmallAirway3 | GSE30063 |
| GSM549795 | NA | NA | 1 | NA | SmallAirway | SmallAirway3 | GSE30063 |
| GSM549796 | NA | NA | 1 | NA | SmallAirway | SmallAirway3 | GSE30063 |
| GSM549797 | NA | NA | 1 | NA | SmallAirway | SmallAirway3 | GSE30063 |
| GSM549798 | NA | NA | 1 | NA | SmallAirway | SmallAirway3 | GSE30063 |
| GSM549799 | NA | NA | 1 | NA | SmallAirway | SmallAirway3 | GSE30063 |
| GSM549800 | NA | NA | 1 | NA | SmallAirway | SmallAirway3 | GSE30063 |
| GSM549801 | NA | NA | 1 | NA | SmallAirway | SmallAirway3 | GSE30063 |
| GSM549802 | NA | NA | 1 | NA | SmallAirway | SmallAirway3 | GSE30063 |
| GSM549803 | NA | NA | 1 | NA | SmallAirway | SmallAirway3 | GSE30063 |
| GSM549804 | NA | NA | 1 | NA | SmallAirway | SmallAirway3 | GSE30063 |
| GSM549805 | NA | NA | 1 | NA | SmallAirway | SmallAirway3 | GSE30063 |
| GSM549806 | NA | NA | 1 | NA | SmallAirway | SmallAirway3 | GSE30063 |
| GSM549807 | NA | NA | 1 | NA | SmallAirway | SmallAirway3 | GSE30063 |
| GSM549808 | NA | NA | 1 | NA | SmallAirway | SmallAirway3 | GSE30063 |
| GSM549809 | NA | NA | 1 | NA | SmallAirway | SmallAirway3 | GSE30063 |
| GSM549810 | NA | NA | 1 | NA | SmallAirway | SmallAirway3 | GSE30063 |
| GSM549811 | NA | NA | 1 | NA | SmallAirway | SmallAirway3 | GSE30063 |
| GSM549812 | NA | NA | 1 | NA | SmallAirway | SmallAirway3 | GSE30063 |
| GSM549813 | NA | NA | 1 | NA | SmallAirway | SmallAirway3 | GSE30063 |
| GSM219241 | 41 | M | 0 | 0 | Alveolar Macrophages | Macrophage1 | GSE8823 |
| GSM219242 | 61 | M | 0 | 0 | AlveolarMacrophages | Macrophage1 | GSE8823 |
| GSM219243 | 37 | F | 0 | 0 | AlveolarMacrophages | Macrophage1 | GSE8823 |
| GSM219244 | 35 | M | 0 | 0 | AlveolarMacrophages | Macrophage1 | GSE8823 |
| GSM219245 | 47 | M | 0 | 0 | AlveolarMacrophages | Macrophage1 | GSE8823 |
| GSM219246 | 36 | M | 0 | 0 | AlveolarMacrophages | Macrophage1 | GSE8823 |
| GSM219247 | 45 | M | 0 | 0 | AlveolarMacrophages | Macrophage1 | GSE8823 |
| GSM219248 | 45 | M | 0 | 0 | AlveolarMacrophages | Macrophage1 | GSE8823 |
| GSM219249 | 49 | F | 0 | 0 | AlveolarMacrophages | Macrophage1 | GSE8823 |
| GSM219250 | 38 | M | 0 | 0 | AlveolarMacrophages | Macrophage1 | GSE8823 |
| GSM219251 | 45 | M | 0 | 0 | AlveolarMacrophages | Macrophage1 | GSE8823 |
| GSM219252 | 37 | F | 1 | 23 | AlveolarMacrophages | Macrophage1 | GSE8823 |
| GSM219253 | 46 | M | 1 | 21 | AlveolarMacrophages | Macrophage1 | GSE8823 |
| GSM219254 | 41 | M | 1 | 20 | AlveolarMacrophages | Macrophage1 | GSE8823 |
| GSM219255 | 44 | F | 1 | 30 | AlveolarMacrophages | Macrophage1 | GSE8823 |
| GSM219256 | 44 | M | 1 | 45 | AlveolarMacrophages | Macrophage1 | GSE8823 |
| GSM219257 | 48 | M | 1 | 15 | AlveolarMacrophages | Macrophage1 | GSE8823 |
| GSM219258 | 40 | M | 1 | 24 | AlveolarMacrophages | Macrophage1 | GSE8823 |
| GSM219259 | 48 | M | 1 | 20 | AlveolarMacrophages | Macrophage1 | GSE8823 |
| GSM219260 | 50 | M | 1 | 38 | AlveolarMacrophages | Macrophage1 | GSE8823 |
| GSM219261 | 45 | M | 1 | 24 | AlveolarMacrophages | Macrophage1 | GSE8823 |
| GSM219262 | 56 | M | 1 | 80 | AlveolarMacrophages | Macrophage1 | GSE8823 |
| GSM219263 | 59 | M | 1 | 60 | AlveolarMacrophages | Macrophage1 | GSE8823 |
| GSM219264 | 45 | M | 1 | 70 | AlveolarMacrophages | Macrophage1 | GSE8823 |
| GSM349912 | 47 | M | 0 | 0 | AlveolarMacrophages | Macrophage2 | GSE13896 |
| GSM349913 | 49 | F | 0 | 0 | AlveolarMacrophages | Macrophage2 | GSE13896 |
| GSM349914 | 34 | M | 0 | 0 | AlveolarMacrophages | Macrophage2 | GSE13896 |
| GSM349915 | 42 | M | 0 | 0 | AlveolarMacrophages | Macrophage2 | GSE13896 |
| GSM349916 | 41 | F | 0 | 0 | AlveolarMacrophages | Macrophage2 | GSE13896 |
| GSM349917 | 35 | M | 0 | 0 | AlveolarMacrophages | Macrophage2 | GSE13896 |
| GSM349918 | 34 | F | 0 | 0 | AlveolarMacrophages | Macrophage2 | GSE13896 |
| GSM349919 | 26 | M | 0 | 0 | AlveolarMacrophages | Macrophage2 | GSE13896 |
| GSM349920 | 24 | M | 0 | 0 | AlveolarMacrophages | Macrophage2 | GSE13896 |
| GSM349921 | 39 | M | 0 | 0 | AlveolarMacrophages | Macrophage2 | GSE13896 |
| GSM349922 | 37 | M | 0 | 0 | AlveolarMacrophages | Macrophage2 | GSE13896 |
| GSM349923 | 29 | M | 0 | 0 | AlveolarMacrophages | Macrophage2 | GSE13896 |
| GSM349924 | 48 | F | 0 | 0 | AlveolarMacrophages | Macrophage2 | GSE13896 |
| GSM349925 | 39 | M | 1 | 30 | AlveolarMacrophages | Macrophage2 | GSE13896 |
| GSM349926 | 41 | M | 1 | 45 | AlveolarMacrophages | Macrophage2 | GSE13896 |
| GSM349927 | 41 | M | 1 | 15 | AlveolarMacrophages | Macrophage2 | GSE13896 |
| GSM349928 | 45 | M | 1 | 23 | AlveolarMacrophages | Macrophage2 | GSE13896 |
| GSM349929 | 50 | F | 1 | 22 | AlveolarMacrophages | Macrophage2 | GSE13896 |
| GSM349930 | 46 | F | 1 | 33 | AlveolarMacrophages | Macrophage2 | GSE13896 |
| GSM349931 | 43 | M | 1 | 30 | AlveolarMacrophages | Macrophage2 | GSE13896 |
| GSM349932 | 36 | M | 1 | 3 | AlveolarMacrophages | Macrophage2 | GSE13896 |
| GSM349933 | 46 | F | 1 | 19 | AlveolarMacrophages | Macrophage2 | GSE13896 |
| GSM349934 | 47 | M | 1 | 11 | AlveolarMacrophages | Macrophage2 | GSE13896 |
| GSM349935 | 41 | M | 1 | 12 | AlveolarMacrophages | Macrophage2 | GSE13896 |
| GSM349936 | 42 | F | 1 | 20 | AlveolarMacrophages | Macrophage2 | GSE13896 |
| GSM349937 | 46 | M | 1 | 26 | AlveolarMacrophages | Macrophage2 | GSE13896 |
| GSM349938 | 32 | F | 1 | 7.6 | AlveolarMacrophages | Macrophage2 | GSE13896 |
| GSM349939 | 27 | F | 1 | 3.8 | AlveolarMacrophages | Macrophage2 | GSE13896 |
| GSM349940 | 35 | M | 1 | 5 | AlveolarMacrophages | Macrophage2 | GSE13896 |
| GSM349941 | 40 | M | 1 | 44.3 | AlveolarMacrophages | Macrophage2 | GSE13896 |
| GSM349942 | 41 | M | 1 | 38 | AlveolarMacrophages | Macrophage2 | GSE13896 |
| GSM349943 | 38 | M | 1 | 20 | AlveolarMacrophages | Macrophage2 | GSE13896 |
| GSM349944 | 27 | F | 1 | 16 | AlveolarMacrophages | Macrophage2 | GSE13896 |
| GSM349945 | 48 | M | 1 | 51 | AlveolarMacrophages | Macrophage2 | GSE13896 |
| GSM349946 | 21 | F | 1 | 20 | AlveolarMacrophages | Macrophage2 | GSE13896 |

*NA: Not Available
